# The Role of Engagement in Virtual Reality to Enhance Emotional Well-Being in Breast Cancer Patients: A Mediation Analysis

**DOI:** 10.3390/cancers17050840

**Published:** 2025-02-28

**Authors:** Hélène Buche, Aude Michel, Royce Anders, Nathalie Blanc

**Affiliations:** 1 Laboratoire Epsylon EA 4556, Université Paul Valéry, 34090 Montpellier, France; royce.anders@univ-montp3.fr (R.A.); nathalie.blanc@univ-montp3.fr (N.B.); 2 Montpellier Institut du Sein, Clinique Clémentville, 34070 Montpellier, France

**Keywords:** virtual reality, sense of presence, engagement, immersion, breast cancer, emotional well-being

## Abstract

Introduction: Virtual reality (VR) has garnered increasing attention in oncology due to its potential to enhance patient care by alleviating anxiety and emotional distress. The present work evaluates the hypothesis proposed by a recent theoretical model that engagement and the sense of presence are key mediators that impact the degree of beneficial effects that VR may have on the emotional well-being of breast cancer patients. Methods: This study draws on data from three previous studies comprising 156 breast cancer patients. The psychological variables of well-being studied included emotional dimensions measured before and after exposure to a virtual environment, as well as factors related to immersive qualities. Correlation and mediation analyses were conducted to explore relationships among said variables, namely, one’s tendency to be immersed in an activity, engagement, spatial presence, and emotional well-being (i.e., valence and arousal) of the patients. Results: Engagement plays a crucial mediating role between tendency of immersion, spatial presence, and positive emotional responses. Patients with a greater tendency toward immersion and higher engagement in the virtual environment showed significant emotional improvements. However, tendency of immersion and spatial presence alone did not directly lead to more positive emotional experiences; their influence was primarily exerted through engagement. Conclusions: Engagement emerges as a fundamental lever for maximizing the psychological benefits of VR in oncology. Clinical interventions using VR should prioritize optimizing engagement in immersive environments to improve patients’ emotional state throughout their cancer treatment journey.

## Simple Summary

This study investigates how virtual reality (VR) can support the emotional well-being of breast cancer patients during their oncology care. Immersing patients in interactive virtual environments has shown potential to reduce anxiety and foster positive emotional states. Our research highlights the central role of engagement in the VR experience, namely, patients’ active cognitive and emotional involvement, as a key factor that amplifies the emotional benefits of immersion. By analyzing data from previous studies, we found that higher levels of engagement significantly improve patients’ emotional states. These findings emphasize the importance of designing VR interventions that actively engage patients, especially during critical stages such as chemotherapy and rehabilitation. This study provides a foundation for developing innovative VR approaches to enhance emotional support in oncology care.

## 1. Introduction

The integration of virtual reality (VR) into cancer care has sparked increasing interest over the past few decades due to its potential to enhance the patient care experience during treatment [1,2,3]. Immersive environments offered by VR have shown their ability to divert attention, reduce anxiety, and improve mood by offering multisensory and interactive experiences [4,5]. This technology has proven particularly effective in creating relaxing environments that can alleviate the emotional burden experienced by patients during oncological treatments [6,7,8]. While scientific research on the benefits of VR in cancer care is particularly abundant today, it is well founded on a long history of VR research and across various sectors or applications [9,10].

A review of the literature by Buche et al. (2022) [1] proposed an integrative theoretical model that provides an explanatory framework for the psychological benefits observed from the use of VR in oncology. This approach highlighted the effects of distractive immersion on improving the emotional state of patients, thus offering a theoretical anchor for the development of future clinical interventions using VR. The theoretical framework is based on key elements: attention resource allocation, multimodality, engagement, and sense of presence, combined with individual characteristics such as patients’ interest in VR and their capacity for engagement [11,12,13,14,15]. According to this model, multimodal immersion naturally draws patients’ attention to pleasant stimuli in the VR environment, actively or passively engaging the patient in the virtual experience [12,16,17]. Engagement refers to the extent to which patients are cognitively and emotionally involved in the virtual environment [14]. Engagement is essential for maximizing the psychological benefits of VR during cancer treatment, helping to keep patients focused on the positive stimuli presented by the virtual system [14,15].

The work of Lessiter et al. (2001) [13] identified engagement as a fundamental sub-factor of the sense of presence, which is conceptualized as a multimodal phenomenon. Presence, often described as “place illusion”, refers to the subjective experience of being transported into the environment represented by the VR interface [18,19,20]. This illusion is closely linked to the sensory, cognitive, and emotional engagement levels of patients [14]. Brockmyer et al. (2009) [21] explored the deep connections between engagement and sense of presence during video game sessions in children, adolescents, and university students. Participants who reported high engagement scores on the Game Engagement Questionnaire [21] demonstrated an increased sense of presence in the interactive application, reflecting deeper integration into the virtual environment.

One’s tendency to become immersed in an activity (hereafter, tendency of immersion), on the other hand, represents an internal factor denoting the capacity to fully engage in an activity, such as a virtual experience, while ignoring external distractions [22,23]. This factor plays a significant role in the perception of presence and the effectiveness of VR interventions [24,25]. In particular, Servotte et al. (2019) [25] evaluated the impact of tendency of immersion and stress on the sense of presence among students exposed to a virtual environment featuring multiple accident victims as part of their academic health care training. The sense of presence was positively correlated with tendency of immersion, meaning that students who had a greater ability to immerse themselves in various situations experienced a higher level of presence. While stress levels increased during immersion, this did not negatively impact their sense of presence [25].

Despite the advantages of VR reported in the literature, the specific roles of presence, engagement, and tendency of immersion in regulating emotional well-being in oncology have not been sufficiently examined or articulated together in a fuller comprehensive theoretical framework [1]. The majority of studies have focused on the direct effects of VR without thoroughly examining the underlying mechanisms that contribute to psychological benefits in the cancer care journey [2,26]. This issue is critical for continuing to advance our understanding of how VR supports the psychological functioning of patients who are, by nature, in a state of vulnerability.

The significance of this article lies in its ambition to offer an integrative perspective on the effects of VR in oncology, bridging both cognitive and emotional dimensions underlying its therapeutic efficacy. While previous research has often adopted a fragmented approach by examining the direct effects of VR without fully articulating the psychological mechanisms involved [2,26], the model proposed here aims to unify the concepts of presence, engagement, and immersion tendency to explain their respective contributions to patients’ emotional well-being. Presence, defined as the subjective illusion of being transported into the virtual environment [18,19,20], lies at the intersection of sensory, cognitive, and affective processes [14]. Engagement, identified as a key factor in the immersive experience [13,21], modulates this presence by determining the depth of users’ integration into the virtual environment [14,15]. Lastly, immersion tendency, which reflects an individual’s ability to ignore external stimuli and fully engage in a virtual activity [22,23], plays a crucial role in the perception of presence and, consequently, in the effectiveness of VR interventions [24,25]. By consolidating these elements within a comprehensive theoretical framework [1], this approach surpasses prior models that have predominantly focused on isolated dimensions, thus providing a deeper understanding of VR’s psychological benefits in oncology.

To understand the associations between presence, engagement, tendency of immersion, and the emotional responses elicited by immersive virtual environments, a mediation analysis was conducted on data collected in three previous studies [6,7,10]. These studies were conducted both during the acute phase of breast cancer treatment (i.e., chemotherapy) and during the post-surgical rehabilitation phase (i.e., physiotherapy), and underscore the necessity of a more holistic approach to patient care. Indeed, different stages of the care pathway present distinct challenges, making it essential to adopt a holistic perspective [27,28,29,30]. In this regard, extending the scope beyond chemotherapy proves particularly valuable, as the majority of existing studies remain confined to this treatment modality, thereby limiting a broader understanding of emotional dynamics across various clinical settings. The findings from this analysis aim to provide a solid foundation for conceptualizing future interventions in care settings to optimize the use of VR. The objective of the present analysis conducted on a full set of three datasets is to establish a robust foundation for the conceptualization of future interventions in care settings, with a view to optimizing the use of VR. Special attention was given to correlations between the sense of presence and immersion tendency, as well as between the ability to engage in a relaxing immersive task and patients’ involvement in their VR experiences, and in the framework of mediation modeling.

Building on theoretical and empirical evidence in immersive technologies, it is hypothesized that engagement in VR should mediate the relationships between both the tendency for immersion and spatial presence with emotional responses. Specifically, a higher tendency for immersion is expected to foster increased engagement in VR, which, in turn, enhances emotional responses, including greater positive emotional valence and reduced arousal levels. However, the tendency for immersion alone is unlikely to directly predict these effects; rather, its influence should be primarily exerted through the mediating role of engagement. Likewise, spatial presence within the virtual environment is anticipated to enhance engagement in VR, thereby amplifying the beneficial effects of immersion on emotional valence and relaxation. Nonetheless, spatial presence alone may not elicit significant emotional benefits; its impact is expected to be mediated by the level of engagement in the VR experience.

In summary, this study seeks to address gaps in the scientific literature by providing empirical evidence of the role of presence and engagement in a relaxing virtual environment to enhance the emotional well-being of women with breast cancer.

## 2. Materials and Methods

### 2.1. Sample

The data analyzed in the present study were selected from three previous studies, two conducted during chemotherapy and a third during post-surgery physiotherapy [6,7,10]. The total sample included 156 patients aged between 29 and 81 years (M = 59.5 years ± 11.17) (see Table 1). None of the selected patients were in the metastatic stage or under palliative care. All the women had breast cancer; the majority of them (*n* = 110) were undergoing treatment at Clinique Clémentville in Montpellier, while a minority (*n* = 46) were in the rehabilitation phase and attending scar massage sessions at a physiotherapy practice associated with the Montpellier Breast Institute (MIS). The inclusion (e.g., read and write in French, be over 18 years old,…) and exclusion criteria (e.g., patients who were pharmacologically treated for anxiety or depression,…) were identical to those applied in the three selected studies [6,7,10].

### 2.2. Materials

In all of the studies used for this mediation analysis [6,7,10], patients experienced the same relaxing virtual environment composed of natural elements. Namely, the *Nature Treks VR* application was used in all three studies due to the well-documented preferences of patients for immersion in natural environments [31,32]. The *Nature Treks VR* application offered immersive environments with diverse natural landscapes, including lush forests, tropical beaches, flower-filled meadows, and underwater scenes, where wildlife and vegetation adapted to user interactions. Patients had the opportunity to choose between passive (i.e., to observe and navigate freely using the controllers) and interactive modes of interaction, the latter allowing them to actively modify certain elements of the environment using a controller, such as making animals appear, changing the sky color, or interacting with the surrounding vegetation. All the measures and associated questionnaires were described in the original research studies [6,7,10]. The comparison across these three studies is facilitated by the consistent use of identical questionnaires and items to assess the variables of interest and to evaluate the same immersive modalities.

The psychological variables studied included emotional dimensions such as valence and arousal, measured using two scales from the *Self-Assessment Manikin* (i.e., *SAM*) [33]. The emotional state data from the three studies showed statistically significant changes between pre- and post-virtual experience, making them suitable for mediation analysis [6,7,10].

Measures of spatial presence and engagement, two crucial indicators of sense of presence in the virtual environment, were assessed using the *ITC-Sense of Presence Inventory* (i.e., *ITC-SOPI*) developed by Lessiter et al. (2001) [13] and translated into French by the Cyberpsychology Laboratory of UQO (2000). Spatial presence assesses the extent to which patients feel “present” in the virtual environment, while engagement measures their level of involvement during immersion. The spatial presence subscale consists of 19 items, whereas the engagement subscale includes 13 items, both rated on a five-point Likert scale.

A variable related to individual predispositions to immersion (i.e., *ITQ*) from the Cyberpsychology Laboratory at UQO (2002) [34] was also utilized, providing insight into patients’ tendencies to immerse themselves in the virtual environment.

Due to the rapid pace of technological advancement, the VR headset used during the first study conducted in physiotherapy [6], the Oculus Go^®^, was replaced by the Meta Quest 2^®^ in the later studies [7,8] in the context of chemotherapy sessions.

### 2.3. Statistical Analyses—Procedure

This study employed a series of correlation and mediation analyses using the software JAMOVI, version 2.3.21, to explore relationships between various variables and the psychological benefits of VR. Descriptive statistics presented in Table 2 summarize the data collected from three studies conducted in oncology [6,7,10] on the impact of VR on patients’ emotional state.

In all three studies, each patient (*n* = 156) used VR during the first 10 min of treatment. Among the 156 participating patients, 96 were exposed to two distinct VR conditions: an active condition involving interaction with the virtual environment and a passive condition in which patients assumed the role as a simple observer. For the remaining 60 patients, they were exposed to only one of these two conditions, with the second session taking place during the following treatment session, either one week later or three weeks later. In the three previous studies [6,7,10], emotional dimensions (i.e., *SAM*) such as valence and arousal were measured before and after exposure to VR. The mean differences in emotional state before and after the immersive experience were calculated and are reported in Table 2.

The *ITC-SOPI* questionnaire was administered immediately after the VR immersion to capture engagement at the moment of experience while the *ITQ* was administered before immersion (see Table 2).

Statistical analyses included paired tests to assess significant differences between pre- and post-VR measures. This approach allowed us to identify the direct impact of the VR intervention on patients. Significant results from these tests are reported in Table 2.

Based on the variables in Table 2, correlation analyses using Pearson’s correlation coefficients were conducted. These analyses assessed the strength and direction of linear relationships between different variables. For example, the relationship between the internal factor of the capacity to engage in a virtual environment (*ITQ* sub-factor) and engagement during the virtual experience (*ITC-SOPI* sub-factor) was examined. Similarly, the correlation between tendency of immersion (*ITQ* total) and sense of presence in the virtual environment (*ITC-SOPI* sub-factor) was examined. These two correlations (*r*) were tested for significance, with a threshold of *p* < 0.05.

In addition to simple correlations, classical mediation analyses were conducted to examine indirect and direct effects between variables using the structural equational modeling framework (SEM). The mediation approach modeled relationships between the tendency of immersion (*ITQ* total), spatial presence (*ITC-SOPI* sub-factor), engagement (*ITC-SOPI* sub-factor), and emotional states (i.e., emotional valence and arousal: *SAM*). Confidence intervals for indirect and total effects were calculated using the Delta method, offering estimates of standard errors and 95% confidence intervals [35].

The classical mediation analysis approach used herein involves the calculation and significance testing for each possible path. That is, the effect of the independent variable on the mediator is estimated; second, the effect of the mediator on the dependent variable is calculated; finally, the direct and total indirect effects of the independent variable on the dependent variable are evaluated. For each of these associations, standardized coefficients were reported to provide a comparable measure of effect sizes between variables.

A priori power analysis was conducted to determine the required sample size for the mediation analysis using G*Power, version 3.1 [36]. The parameters included a medium effect size of 0.15, a significance criterion of 0.05, and a power level of 0.80. For the mediation analysis (i.e., *SAM valence*, *SAM arousal*), the minimum required sample size was *n* = 68. This theoretical sample size was largely satisfied by the actual sample size utilized in our analysis, comprising *n* = 156 participants.

These statistical considerations ensured a thorough and reliable exploration of the dynamics between tendency of immersion, engagement, spatial presence, and emotional responses in a relaxing virtual environment.

## 3. Results

The analysis of the three studies allowed for the examination of variations in psychological variables before and after exposure to VR. Table 2 below presents the average differences observed for each psychological dimension as well as scores obtained before and after the VR experience. These data illustrate the impact of the immersive experience on participants’ emotional states, sense of presence, and tendency of immersion.

The results reveal significant changes before and after VR. Specifically, the data show a notable improvement in emotional valence after the immersive experience, indicating a shift toward a more positive emotional state. The decrease in arousal suggests a potential state of relaxation induced by immersion.

In terms of presence, the scores for spatial presence and engagement measured after immersion indicate a high level of immersion experienced by patients in the virtual environment. Additionally, the high scores on the *ITQ* suggest a strong tendency of immersion, indicating a high capacity for patients to engage in an interactive and immersive experience.

### 3.1. Correlations

#### 3.1.1. Capacity for Engagement and Immersion Engagement

The Pearson correlation analysis reveals a significant positive relationship between the tendency to engage in an immersive task and the level of engagement in VR (*r* = 0.17, *p* = 0.004) with a weak degree of association. Although modest, this suggests that patients with a higher capacity for engagement are slightly more likely to show increased engagement in VR.

#### 3.1.2. Spatial Presence and Tendency of Immersion

The Pearson correlation analysis also showed a significant positive association between spatial presence in a virtual environment and the tendency to be immersed in an activity (*r* = 0.23, *p* < 0.001), also with a weak-to-moderate degree of association. This suggests that individuals with greater immersion ability tend to experience higher spatial presence in VR.

### 3.2. Mediation

#### 3.2.1. Impact of Tendency of Immersion via Engagement on Emotional Valence

**Figure 1:**
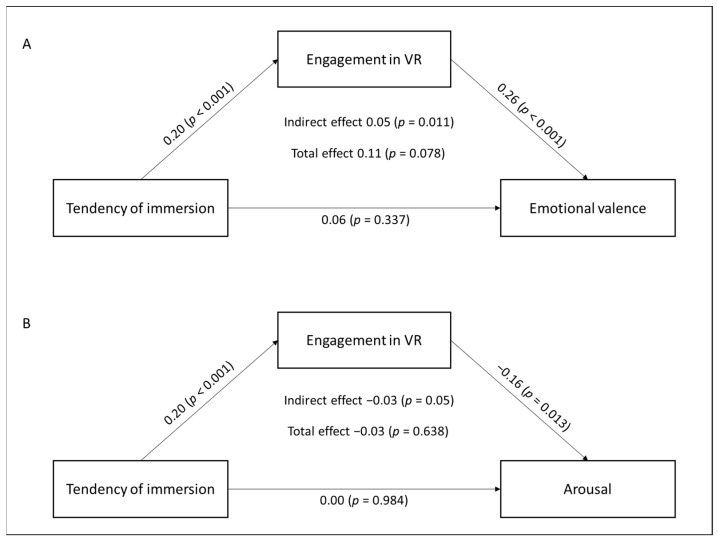
Mediation Model of Engagement in VR: Direct and Indirect Effects, Tendency of Immersion on Emotional Valence (**A**) and Arousal (**B**).

This mediation analysis examines the link between tendency of immersion and the induction of positive emotional responses, considering engagement during virtual immersion as a potential mediator. Positive emotional responses are measured by the difference in emotional valence between pre- and post-VR exposure (see Table 2 and Figure 1A).

The results of the analysis show that the indirect effect of tendency of immersion on emotional valence, mediated by engagement, is statistically significant (*β* = 0.05, 95% CI [0.00, 0.00], *p* = 0.011). Patients’ immersion tendency can predict the induction of more positive emotional responses after a virtual reality experience, mediated by high levels of engagement in the environment.

The significant direct effect of tendency of immersion on engagement (*β* = 0.20, 95% CI [0.00, 0.01], *p* = 0.001) indicates that greater immersion tendency is strongly associated with higher engagement in VR. Furthermore, the significant direct effect of engagement on emotional valence (*β* = 0.26, 95% CI [0.38, 1.04], *p* < 0.001) suggests that greater engagement in the virtual environment is linked to more positive emotional experiences during VR. However, the direct effect of tendency of immersion on emotional valence is not statistically significant (*β* = 0.06, 95% CI [−0.00, 0.01], *p* = 0.337). Tendency of immersion does not have a direct impact on the induction of more positive emotional responses in VR.

These results highlight the importance of engagement as a mediator, suggesting that tendency of immersion can predict high engagement levels in the virtual environment and, through this pathway, positively influence emotional valence.

#### 3.2.2. Impact of Tendency of Immersion via Engagement on Arousal Level

This mediation analysis examines the association between tendency of immersion and changes in arousal levels, considering engagement as a potential mediator. Emotional relaxation is measured by the mean difference in arousal levels between before and after the VR session (see Table 2 and Figure 1B).

The results show that the indirect effect of tendency of immersion on arousal, mediated by engagement, suggests significance (*β* = −0.03, 95% CI [−0.00, 0.00], *p* = 0.05). Engagement plays a mediating role in the association between tendency of immersion and emotional relaxation after VR. The significant direct effect of tendency of immersion on engagement (*β* = 0.20, 95% CI [0.00, 0.01], *p* = 0.001) indicates that a greater tendency of immersion is associated with a higher level of engagement. Furthermore, the direct effect of engagement on arousal is also significant (*β* = −0.16, 95% CI [−0.94, −0.11], *p* = 0.013). Higher engagement in the VR session is linked to greater emotional relaxation during oncology care. However, the direct effect of tendency of immersion on arousal is not statistically significant (*β* = 0.00, 95% CI [−0.02, 0.02], *p* = 0.984). Consistent with this, tendency of immersion does not directly impact the reduction of emotional tension.

This analysis underscores engagement as a key mediator between tendency of immersion and arousal levels. Although tendency of immersion has no direct effect on emotional relaxation, engagement mediates this relationship, highlighting its importance in VR use.

#### 3.2.3. Impact of Spatial Presence via Engagement on Emotional Valence

**Figure 2:**
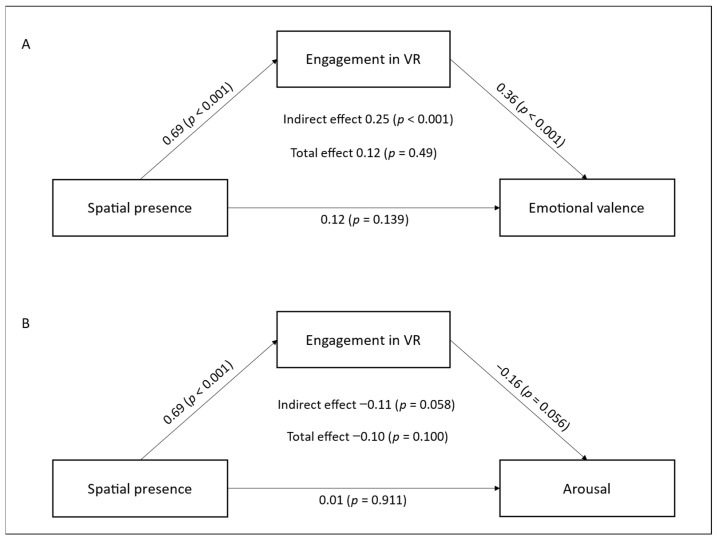
Mediation Model of Engagement in VR: Direct and Indirect Effects, Spatial Presence on Emotional Valence (**A**) and Arousal (**B**).

This mediation examines the indirect effects of engagement in immersive tasks and the direct effects of spatial presence in a virtual environment on inducing a more positive emotional state in breast cancer patients. Positive emotional response is measured by the difference in valence before and after the VR session (see Table 2 and Figure 2A).

The analysis shows a statistically significant indirect effect of spatial presence on emotional valence, mediated by engagement (*β* = 0.25, 95% CI [0.30, 0.85], *p* < 0.001). Spatial presence influences changes in emotional valence primarily through engagement in the virtual environment. The significant direct effect of spatial presence on engagement (*β* = 0.69, 95% CI [0.51, 0.67], *p* < 0.001) indicates that a stronger sense of spatial presence is linked to higher engagement in VR. Similarly, the significant direct effect of engagement on emotional valence (*β* = 0.36, 95% CI [0.53, 1.42], *p* < 0.001) indicates that higher engagement is related to more positive emotional experiences following VR immersion. However, the direct effect of spatial presence on emotional valence is not statistically significant (*β* = 0.12, 95% CI [−0.67, 0.09], *p* = 0.139). When controlling for engagement, spatial presence has no direct impact on inducing more positive emotional experiences.

Finally, the total effect of spatial presence on emotional valence is significant (*β* = 0.12, 95% CI [0.00, 0.58], *p* = 0.049). Although predominantly mediated by engagement, spatial presence nonetheless plays a contributory role in eliciting a more positive emotional response following virtual immersion.

These findings underscore engagement as a mediator between spatial presence and improved emotional well-being in VR. Spatial presence enhances positive emotions mainly by increasing engagement in immersive tasks.

#### 3.2.4. Impact of Spatial Presence via Engagement on Arousal

This mediation explores the indirect effects of engagement in an immersive task and the direct effects of spatial presence experienced in a virtual environment on the reduction of emotional tension in patients. Emotional relaxation is measured by the mean difference in arousal levels between before and after the VR session (see Table 2, Figure 2B).

The results indicate that the indirect effect of spatial presence on arousal, mediated by engagement, is not significant (*β* = −0.11, 95% CI [−0.66, 0.01], *p* = 0.058). However, a trend emerges, suggesting that spatial presence may help alleviate emotional tension following virtual immersion through the mediating role of engagement. Additionally, the direct effect of spatial presence on engagement is significant (*β* = 0.69, 95% CI [0.52, 0.67], *p* < 0.001). A strong sense of spatial presence is associated with a higher level of engagement during the immersion. Regarding the direct effect of engagement on arousal, it is only marginally significant (*β* = −0.16, 95% CI [−1.11, 0.01], *p* = 0.056). These findings imply that greater engagement may foster a reduction in emotional tension, albeit with a magnitude insufficient to meet strict significance thresholds. Moreover, the direct effect of spatial presence on the reduction of emotional tension is not significant (*β* = 0.01, 95% CI [−0.46, 0.51], *p* = 0.911). Spatial presence alone does not have a direct impact on this variable.

These results highlight the importance of engagement in immersive virtual environments, which represents a potential mechanism for inducing emotional relaxation.

## 4. Discussion

The aim of this study was to explore how the sense of presence, engagement, and tendency for immersion contribute to emotional well-being in breast cancer patients during oncology care through VR sessions. These findings are also applied to the theoretical model proposed by Buche et al. (2022) [1] to clarify how immersive factors impact emotional states, focusing on critical phases such as chemotherapy and post-surgical rehabilitation.

The theoretical model highlights the role of attention allocation, engagement, and presence in improving emotional states through VR. This study supports their relevance by showing that engagement mediates the relationship between involvement and emotional responses. Consistent with Lessiter et al. (2001) [13] and Garrett et al. (2020) [14], engagement also mediates between tendency for immersion and emotional outcomes, most predominantly with regard to emotional valence.

Correlation analyses revealed modest but significant associations between involvement, engagement, and spatial presence, validating the key role of cognitive and emotional engagement in enhancing VR effectiveness. Mediation analyses further revealed that engagement amplifies the emotional benefits of VR, as noted by Brockmyer et al. (2009) [21], who linked immersive impact to engagement.

Spatial presence contributes to emotional valence, yet its effect is mediated by engagement, which plays a decisive role in fostering well-being. While presence alone remains insufficient, engagement driven by spatial presence emerges as the primary mechanism underlying immersive emotional benefits. Moreover, spatial presence tends to influence arousal, though its impact is engagement dependent and associated with a small effect size. This aligns with findings by Bouchard et al. (2008) [18] and Slater et al. (2022) [20], showing that greater engagement in VR leads to more positive emotions.

In sum, engagement is the key mechanism translating immersive VR experiences into emotional benefits, emphasizing the importance of designing highly engaging VR environments to optimize psychological outcomes for oncology patients.

### 4.1. Empirically Enriched Theoretical Model

**Figure 3:**
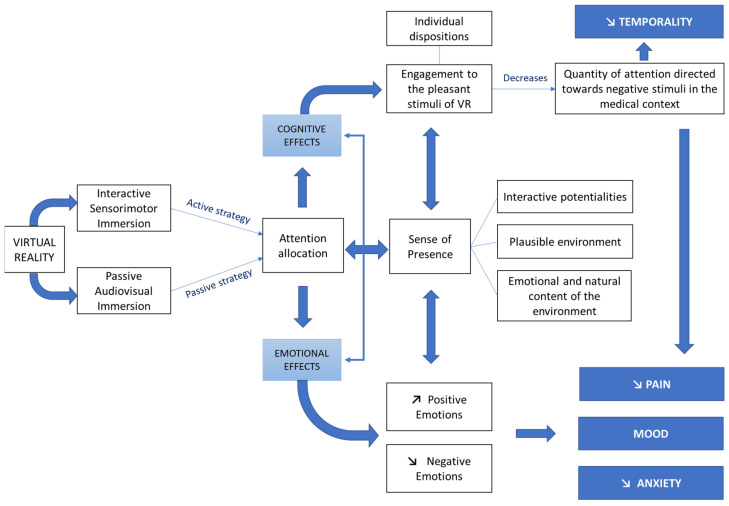
Enriched Theoretical Model Derived From Mediation Analyses That Highlighted Interactive Potentialities.

A comprehensive theoretical model (see Figure 3), based on empirical works, has been proposed [1] to highlight the cognitive and emotional mechanisms through which VR may improve the mental well-being of cancer patients. The bidirectional interaction between cognitive and emotional effects underscores the interdependent nature of these processes, which collectively shape the patient’s overall experience in the immersive environment. These cognitive and emotional modulations do not function in isolation but rather operate synergistically, thereby influencing the therapeutic outcomes of VR interventions [37]. While this framework provides a solid explanatory basis, it does not fully account for the mediating role of engagement in converting immersive properties into emotional benefits. Addressing this gap, the present study refines and extends the model, demonstrating that spatial presence alone is insufficient to induce significant emotional well-being. Contrary to the direct and bidirectional association posited by Riva et al. (2007) [38], our findings show that its effect is primarily mediated by engagement, which plays a crucial role in immersive processes.

Based on the mediation modeling results herein, the model may be refined in several key respects. First, engagement emerges as the central mechanism mediating the relationship between presence and emotional well-being, highlighting its crucial role in translating immersive properties into emotional benefits. Second, dispositional traits such as capacity for involvement, interest, and immersion tendency are identified as key determinants shaping engagement, reinforcing its impact on emotional outcomes. Third, this enriched model strengthens the conceptual foundation by positioning engagement as the core of immersive dynamics, suggesting that a bidirectional relationship between presence and engagement could better reflect this dynamic.

These refinements allow for a more precise articulation of the causal pathways through which VR fosters emotional well-being in oncology patients. Specifically, they highlight (a) the necessity of fostering engagement to optimize immersive benefits, (b) the contribution of individual predispositions in shaping engagement levels, and (c) the interactive mechanisms that underpin the emotional efficacy of VR interventions. Importantly, this enriched model provides a more nuanced conceptual foundation for the design of VR environments aimed at maximizing patient engagement and, consequently, their psychological benefits.

Future research should explore in greater depth the mechanisms through which engagement can be enhanced, particularly, through personalization strategies that tailor virtual environments to patients’ individual preferences and responses. This could include the adaptation of content and interactivity based on physiological or behavioral feedback, allowing for dynamic adjustments that optimize engagement. Integrating familiar or emotionally resonant stimuli, alongside refining interactive elements and the plausibility of the virtual environment, would be essential for improving immersive interventions in clinical settings and ensuring their long-term efficacy.

### 4.2. Limitations

This study has several limitations. First, variability in VR devices, such as the Oculus Go^®^ and Meta Quest 2^®^, may introduce heterogeneity in immersive experiences, potentially affecting results replication. Similarly, assessing patients’ emotional states in different care contexts (i.e., chemotherapy and post-surgical rehabilitation) may introduce contextual bias, as emotional distress and expectations vary over time and across clinical situations [27,28,29,30]. Nonetheless, VR was effective at both care pathway stages [6,7].

Additionally, the patient sample used in the analysis herein constituted a sample of individuals that represents the different phases of the cancer treatment continuum (stages of chemotherapy and rehabilitation). While this allowed for detection of the general trends that virtual reality variables could have on engagement and emotional well-being, in the optics of a first study assessing this question, it is important to note the essential need for further work on this topic, analyzing in greater depth the potential interactions that may arise based on treatment phase and its degree of success (e.g., chemotherapy). Therefore, with an appropriate sample size by group (and progress within), it is possible that when controlling for these variables, the trends in the mediation analyses found herein may potentially be modified, highlighting the contributive value of continuing this line of research. A larger patient sample would have been preferable, despite power analyses confirming the present sample’s adequacy. Recruiting a large sample in clinical applications remains challenging but could add value to the current results.

Moreover, the study relied on self-reported measures for emotional assessment, which are inherently susceptible to subjective biases. To enhance the robustness of future investigations, it would be pertinent to complement self-reports with physiological or behavioral indices, thereby providing a more comprehensive and objective appraisal of emotional states [17].

Lastly, the study did not explore dynamic interactions between engagement and emotional experience over time, warranting future longitudinal research [8]. The emotional benefits of VR may partly result from a novelty effect, especially in patients unfamiliar with the technology [6]. Long-term studies should examine these evolving benefits and the interaction of psychological variables in different oncology care settings [15].

### 4.3. Perspectives

One of the main strengths of this study is that it opens up a reflection on the individual characteristics related to patients’ cognitive and emotional engagement during their care pathway, such as resilience, self-efficacy, or even self-compassion, which could enhance the understanding of VR’s effects [39,40] and ultimately lead to more effective outcomes.

Although the plausibility of the virtual environment was not directly measured in this study, it emerges as a relevant theoretical perspective for future research. Plausibility, by enhancing the perceived credibility of the virtual environment, could serve as an additional catalyst for emotional engagement and the sense of presence, thereby amplifying the positive effects of VR on patients’ well-being [1,20]. This concept warrants particular attention in the design of immersive interventions, especially in clinical settings, where the perceived realism of the environment can play a key role in patients’ emotional investment.

Finally, this study’s results are complementary and corroborate the validity of the theoretical model by Buche et al. (2022) [1] and pave the way for deeper reflection on the use of immersive technologies in oncology, contributing to a foundation of innovative, more targeted, engaging, and potentially transformative clinical interventions to support cancer patients.

## 5. Conclusions

This study highlights the central role of engagement in the beneficial effects of VR on the emotional well-being of breast cancer patients. While tendency for immersion and spatial presence are essential, their impact on emotional state is primarily indirect, mediated through active engagement. These findings underscore the importance of designing VR interventions that foster deep engagement, particularly by enhancing spatial presence, to maximize emotional benefits. Optimizing engagement, and taking into account personal dispositions (proneness to immersion), thus paves the way for personalized clinical interventions tailored to patients’ needs throughout their care pathway.

## Figures and Tables

**Table 1:** Characteristics of the Selected Sample.

Variables	Participants	%
Age: Mean (SD)	59.5 (11.17)	
Marital status		
Married/couple	*N* = 97	62.18
Single/separated/divorced	*N* = 49	37.82
Employed		
Yes	*N* = 48	30.77
No	*N* = 108	69.23
SD, Standard Deviation		

**Table 2:** Descriptive Data of Psychological Variables in the Three Studies.

Psychological Variables	Before VR Mean (SD)	After VR Mean (SD)	Mean Difference (SD)
**Mood state**			
Valence	6.67 (2.04)	7.93 (1.46)	1.26 (1.75) ***
Arousal	3.68 (2.33)	2.29 (1.74)	−1.39 (2.14) ***
**Sense of presence**			
Spatial presence	-	3.57 (0.75)	-
Engagement	-	3.90 (0.64)	-
**Tendency of immersion**			
Focus	25.04 (4.96)	-	-
Engagement	20.41 (7.12)	-	-
Emotion	16.74 (5.91)	-	-
Games	8.08 (4.52)	-	-
Total	74.90 (17.10)	-	-

SD, Standard Deviation. ***, *p* < 0.001.

## Data Availability

The raw data supporting the conclusions of this article will be made available by the authors, without undue reservation.

## References

[1] Buche H., Michel A., Blanc N. (2022). Use of virtual reality in oncology: From the state of the art to an integrative model. Front. Virtual Real..

[2] Mingqin L., Yuting S., Yushuo N., Ting L., Song G.E., Yaru S., Xin W., Ying L., Kuinan L., Xiuling Y. (2024). Effectiveness of Virtual Reality in the Management of Anxiety and Pain Peri-Treatment for Breast Cancer: A Systematic Review and Meta-Analysis. J. Nurs. Res..

[3] Sezgin M.G., Bektas H. (2024). Research Trends and Highlights Toward Virtual Reality in Patients With Cancer: Bibliometric Analysis. CIN Comput. Inform. Nurs..

[4] Pardini S., Gabrielli S., Dianti M., Novara C., Zucco G.M., Mich O., Forti S. (2022). The role of personalization in the user experience, preferences and engagement with virtual reality environments for relaxation. Int. J. Environ. Res. Public Health.

[5] Wang Z., Li Y., An J., Dong W., Li H., Ma H., Wang J., Wu J., Jiang T., Wang G. (2022). Effects of restorative environment and presence on anxiety and depression based on interactive virtual reality scenarios. Int. J. Environ. Res. Public Health.

[6] Buche H., Michel A., Piccoli C., Blanc N. (2021). Contemplating or acting? Which immersive modes should be favored in virtual reality during physiotherapy for breast cancer rehabilitation. Front. Psychol..

[7] Buche H., Michel A., Blanc N. (2023). When virtual reality supports patients’ emotional management in chemotherapy. Front. Virtual Real..

[8] Sharma A., Sharma N., Chahal A. (2024). Impact of Virtual Reality on Pain, ROM, Muscle Strength and Quality of Life among Breast Cancer Patients: An Integrative Review of Literature. Pain Manag. Nurs. Off. J. Am. Soc. Pain Manag. Nurses.

[9] Buche H. (2024). Comprendre et Expliquer les Effets de la Réalité Virtuelle à Différentes Etapes de la Prise en Charge du Cancer du Sein: Apports Théoriques et Empiriques. Ph.D. Thesis.

[10] Buche H., Michel A., Blanc N. Using Virtual Reality During Chemotherapy to Support Emotional Regulation in Patients: Adding an Olfactory Reinforcement or not?. (under review).

[11] Arane K., Behboudi A., Goldman R.D. (2017). Virtual reality for pain and anxiety management in children. Can. Fam. Physician.

[12] Chirico A., Maiorano P., Indovina P., Milanese C., Giordano G.G., Alivernini F., Iodice G., Gallo L., De Pietro G., Lucidi F. (2020). Virtual reality and music therapy as distraction interventions to alleviate anxiety and improve mood states in breast cancer patients during chemotherapy. J. Cell. Physiol..

[13] Lessiter J., Freeman J., Keogh E., Davidoff J. (2001). A cross-media presence questionnaire: The ITC-sense of presence inventory. Presence Teleoperators Virtual Environ..

[14] Garrett B.M., Tao G., Taverner T., Cordingley E., Sun C. (2020). Patients perceptions of virtual reality therapy in the management of chronic cancer pain. Heliyon.

[15] Burrai F., Sguanci M., Petrucci G., De Marinis M.G., Piredda M. (2023). Effectiveness of immersive virtual reality on anxiety, fatigue and pain in patients with cancer undergoing chemotherapy: A systematic review and meta-analysis. Eur. J. Oncol. Nurs. Off. J. Eur. Oncol. Nurs. Soc..

[16] Ahmadpour N., Keep M., Janssen A., Rouf A.S., Marthick M. (2020). Design strategies for virtual reality interventions for managing pain and anxiety in children and adolescents: Scoping review. JMIR Serious Games.

[17] Erdős S., Horváth K. (2023). The Impact of Virtual Reality (VR) on Psychological and Physiological Variables in Children Receiving Chemotherapy: A Pilot Cross-Over Study. Integr. Cancer Ther..

[18] Bouchard S., St-Jacques J., Robillard G., Renaud P. (2008). Anxiety increases the feeling of presence in virtual reality. Presence Teleoperators Virtual Environ..

[19] Baus O., Bouchard S. (2014). Moving from virtual reality exposure-based therapy to augmented reality exposure-based therapy: A review. Front. Hum. Neurosci..

[20] Slater M., Banakou D., Beacco A., Gallego J., Macia-Varela F., Oliva R. (2022). A separate reality: An update on place illusion and plausibility in virtual reality. Front. Virtual Real..

[21] Brockmyer J.H., Fox C.M., Curtiss K.A., McBroom E., Burkhart K.M., Pidruzny J.N. (2009). The development of the Game Engagement Questionnaire: A measure of engagement in video game-playing. J. Exp. Soc. Psychol..

[22] Robillard G., Bouchard S., Renaud P., Cournoyer L.G. Validation canadienne-française de deux mesures importantes en réalité virtuelle: L’Immersive Tendencies Questionnaire et le Presence Questionnaire. Proceedings of the 25e Congrès Annuel de la Société Québécoise Pour la Recherche en Psychologie (SQRP).

[23] Wallach H.S., Safir M.P., Samana R. (2010). Personality variables and presence. Virtual Real..

[24] Kober S.E., Neuper C. (2013). Personality and presence in virtual reality: Does their relationship depend on the used presence measure?. Int. J. Humancomput. Interact..

[25] Servotte J.C., Goosse M., Campbell S.H., Dardenne N., Pilote B., Simoneau I.L., Guillaume M., Bragard I., Ghuysen A. (2020). Virtual reality experience: Immersion, sense of presence, and cybersickness. Clin. Simul. Nurs..

[26] Hao J., Li Y., Swanson R., Chen Z., Siu K.C. (2023). Effects of virtual reality on physical, cognitive, and psychological outcomes in cancer rehabilitation: A systematic review and meta-analysis. Support. Care Cancer.

[27] Helgeson V.S., Snyder P., Seltman H. (2004). Psychological and physical adjustment to breast cancer over 4 years: Identifying distinct trajectories of change. Health Psychol..

[28] Deshields T., Tibbs T., Fan M.Y., Taylor M. (2006). Differences in patterns of depression after treatment for breast cancer. Psycho-Oncol. J. Psychol. Soc. Behav. Dimens. Cancer.

[29] Lam W.W., Bonanno G.A., Mancini A.D., Ho S., Chan M., Hung W.K., Or A., Fielding R. (2010). Trajectories of psychological distress among Chinese women diagnosed with breast cancer. Psycho-Oncol..

[30] Zhang Y., Yan J., He H., Zhang L., Chen L., Li N., Li H., Zhang L., Zhang N., Sun S. (2024). The trajectories of psychosocial adjustment among young to middle-aged women with breast cancer: A prospective longitudinal study. Eur. J. Oncol. Nurs..

[31] Michel A., Vidal J., Brigaud E., Sokratous K., Blanc N. (2019). Dessine-moi une réalité plus belle: La réalité virtuelle vue par les patients atteintes d’un cancer du sein. Psycho-Oncologie.

[32] Michel A., Brigaud E., Cousson-Gélie F., Vidal J., Blanc N. (2019). La réalité virtuelle chez les femmes âgées suivies pour un cancer du sein: Intérêts et attentes. Gériatrie Psychol. Neuropsychiatr. Vieil..

[33] Bradley M.M., Lang P.J. (1994). Measuring emotion: The self-evaluation dummy and the semantic differential. J. Behav. Ther. Exp. Psychiatry.

[34] Cyberpsychology Laboratory of UQO (2002). Cyberpsychologie. http://w3.uqo.ca/cyberpsy/index.php/documents-utiles/.

[35] Oehlert G.W. (1992). A Note on the Delta Method. Am. Stat..

[36] Faul F., Erdfelder E., Lang A.G., Buchner A. (2007). G* Power 3: A flexible statistical power analysis program for the social, behavioral, and biomedical sciences. Behav. Res. Methods.

[37] Bouvier P. (2009). La Présence en Réalité Virtuelle, une Approche Centrée Utilisateur. Ph.D. Thesis.

[38] Riva G., Mantovani F., Capideville C.S., Preziosa A., Morganti F., Villani D., Gaggioli A., Botella C., Alcañiz M. (2007). Affective interactions using virtual reality: The link between presence and emotions. Cyberpsychol. Behav. Impact Internet Multimed. Virtual Real. Behav. Soc..

[39] Kelleher S.A., Fisher H.M., Winger J.G., Miller S.N., Amaden G.H., Somers T.J., Colloca L., Uronis H.E., Keefe F.J. (2022). Virtual reality for improving pain and pain-related symptoms in patients with advanced stage colorectal cancer: A pilot trial to test feasibility and acceptability. Palliat. Support. Care.

[40] O’Gara G., Murray L., Georgopoulou S., Anstiss T., Macquarrie A., Wheatstone P., Bellman B., Gilbert P., Steed A., Wiseman T. (2022). SafeSpace: What is the feasibility and acceptability of a codesigned virtual reality intervention, incorporating compassionate mind training, to support people undergoing cancer treatment in a clinical setting?. BMJ Open.

